# Is elective inguinal radiotherapy necessary for locally advanced rectal adenocarcinoma invading anal canal?

**DOI:** 10.1186/s13014-014-0296-1

**Published:** 2014-12-23

**Authors:** Seung-Gu Yeo, Hyeon Woo Lim, Dae Yong Kim, Tae Hyun Kim, Sun Young Kim, Ji Yeon Baek, Hee Jin Chang, Ji Won Park, Jae Hwan Oh

**Affiliations:** Department of Radiation Oncology, Soonchunhyang University College of Medicine, Choenan, Korea; Proton Therapy Center, Research Institute and Hospital, National Cancer Center, Goyang, Korea; Center for Colorectal Cancer, Research Institute and Hospital, National Cancer Center, Goyang, Korea

**Keywords:** Rectal cancer, Radiotherapy, Inguinal lymph node, Anal canal invasion

## Abstract

**Background:**

We investigated whether routine elective irradiation of a clinically negative inguinal node (IGN) is necessary for patients with locally advanced distal rectal cancer and anal canal invasion (ACI).

**Methods:**

We reviewed retrospectively 1,246 patients with locally advanced rectal adenocarcinoma managed using preoperative or postoperative chemoradiotherapy and radical surgery between 2001 and 2011. The patients’ IGN was clinically negative at presentation and IGN irradiation was not performed. ACI was defined as the lower edge of the tumor being within 3 cm of the anal verge. Patients were divided into two groups, those with ACI (n = 189, 15.2%) and without ACI (n = 1,057, 84.8%).

**Results:**

The follow-up period was a median of 66 months (range, 3–142 months). Among the 1,246 patients, 10 developed IGN recurrence; 7 with ACI and 3 without ACI. The actuarial IGN recurrence rate at 5 years was 0.7%; 3.5% and 0.2% in patients with and without ACI, respectively (*p* < 0.001). Isolated IGN recurrence occurred in three patients, all of whom had ACI tumors. These three patients received curative intent local treatments, and one was alive with no evidence of disease 10 years after IGN recurrence. Salvage treatments in the other two patients controlled successfully the IGN recurrence for >5 years, but they developed second malignancy or pelvic and distant recurrences. Seven patients with non-isolated IGN recurrence died of disease at 5–22 months after IGN recurrence.

**Conclusion:**

The low IGN recurrence rate even with ACI and the feasibility of salvage of isolated IGN recurrence indicated that routine elective IGN irradiation is not necessary for rectal cancer with ACI.

## Background

The lymphatic drainage from the rectum occurs preferentially in a proximal direction. The lymphatic system supplies the mesenteric and para-aortic nodes through the perirectal and pelvic lymphatic vessels. However, when a distal rectal tumor invades the anal canal, the lymphatic system drains in another direction to the inguinal nodes (IGN), as observed in primary anal cancer, through the perirectal, perianal and pudendal lymphatics [1].

Radiation therapy (RT) is an important constituent of multi-modality management recommended for locally advanced (stage II or III) rectal cancer [2]. Preoperative or postoperative RT reduces local disease recurrence and the former improves resectability and sphincter preservation by downstaging or downsizing the tumor [3]. Conventional RT fields for rectal cancer encompass regional lymphatics which do not contain IGN [4], whereas standard RT fields for anal cancer involve IGN because the risk of local recurrence is high in this region without elective treatment [5]. Thus, for a subset of rectal cancer patients with anal canal invasion (ACI) and with potential lymphatic drainage to IGN, whether RT fields should be extended to involve IGN basins remains questionable [5].

In our institution, the treatment policy for rectal cancer with clinically negative IGN at presentation has been no irradiation of IGN, regardless of tumor location. To determine the requirement for routine elective IGN irradiation for patients with distal rectal cancer and ACI, we investigated the failure patterns and salvage feasibility regarding IGN recurrence and conducted a comparison between rectal tumors with and without ACI.

## Methods

### Patients

A total of 1,246 patients with locally advanced rectal cancer treated with preoperative or postoperative chemoradiotherapy (CRT) and radical surgery between 2001 and 2011 at the National Cancer Center (Goyang, Korea) were reviewed retrospectively. The patients had histologically confirmed rectal adenocarcinoma and no evidence of distant metastasis, including IGN, at the time of initial workup and surgery. Concurrent CRT was administered preoperatively (cT3–4) or postoperatively (pT3–4 or pN+) in 877 (70.4%) and 369 (29.6%) patients, respectively. Since the introduction of preoperative CRT at our institution in October 2001, routine treatment for clinically staged T3–4 rectal cancer located at the mid-to-low rectum (≤9 cm from the anal verge) has gradually changed from postoperative to preoperative CRT. During and after the transition period, upfront surgery with postoperative CRT was performed routinely for upper rectal cancer, and for mid-to-low rectal cancer, it was determined by the preferences of patients or attending physicians.

Pretreatment workups for clinical staging included digital rectal examination, complete blood count, liver function tests, serum carcinoembryonic antigen tests, video colonoscopy, chest radiography and computed tomography (CT) scanning of the abdomen and pelvis with or without transrectal ultrasonography and pelvic magnetic resonance imaging (MRI). ^18^F-deoxyfluoroglucose positron emission tomography-CT was performed as required. Clinically positive lymph node involvement was defined as a lymph node with the smallest diameter of 0.5 cm, observed on CT or MRI. All stages were determined according to the American Joint Committee on Cancer Staging System, 7th edition [4]. Anal canal involvement (ACI) was defined when the lower edge of the tumor was within 3 cm from the anal verge on colonoscopy or digital rectal examination. Digital rectal examination for all patients in the present study was performed by one experienced radiation oncologist (Dr. DY Kim). Patients were grouped and compared according to the existence of ACI; 189 (15.2%) with ACI and 1,057 (84.8%) without ACI. All patients provided written informed consent before treatments and the study was performed in accordance with the guidelines of our Institutional Review Board of the National Cancer Center.

### Treatments

Radiotherapy was delivered to the whole pelvis at a dose of 45 Gy in 25 fractions, followed by a 5.4-Gy boost in three fractions within 6 weeks. All patients underwent CT simulation for three-dimensional conformal planning, and a three-field treatment plan used a 6-MV photon posterior-anterior field and 15-MV photon-opposed lateral beams. The prescription dose was specified at the isocenter of the planning target volume. The initial radiation field encompassed the gross tumor and mesorectum (preoperative CRT) or tumor bed (postoperative CRT), presacral space, the entire sacral hollow and the regional lymphatics, including the perirectal, internal iliac, presacral and distal common iliac lymphatics. The superior border was placed at L5/S1 and the inferior border at > 3 cm caudal to the gross tumor or tumor bed. The boost field included the gross tumor volume and mesorectum (preoperative CRT) or tumor bed (postoperative CRT), with ≥ 2 cm margin in all directions.

Chemotherapy administered concurrently with RT used one of the following regimens: fluoropyrimidine, irinotecan, or an oxaliplatin-based regimen. Patients underwent radical proctectomy, including high ligation of the inferior mesenteric vessels and total mesorectal excision. The interval between preoperative CRT and surgery was 4–8 weeks, and 3–8 weeks between surgery and postoperative CRT. Postoperative chemotherapy was initiated 3–6 weeks after surgery or postoperative CRT, using fluoropyrimidine or an oxaliplatin-based regimen.

### Evaluation

All patients underwent standardized follow-up, consisting of physical examination, complete blood count, liver function tests, serum carcinoembryonic antigen tests and chest radiography every 3 months for the first 2 years and every 6 months thereafter, as well as abdominopelvic CT every 6 months. Colonoscopic examinations were performed 1 year postoperatively and then at 2-year intervals. Recurrence was determined based on clinical, radiological or histological findings. Radiological evidence involved serial radiological examinations showing progressive mass growth, including abnormally high uptake on ^18^F-deoxyfluoroglucose positron emission tomography.

### Analysis

Intergroup comparisons regarding patient, tumor and treatment characteristics were conducted using the chi-square test, Fisher’s exact test, linear-by-linear association, or *t-*test, depending on the nature of the data. Multivariate logistic regression was conducted to determine parameters that are related with IGN recurrence. Actuarial IGN recurrence rate was estimated using the Kaplan-Meier method and the significance of differences was assessed with the log-rank test. The level of statistical significance was set at *p* < 0.05 and all reported *p*-values were two-tailed. Statistical analyses were performed using the SPSS software (version 14.0; SPSS Inc., Chicago, IL, USA).

## Results

### Patients

Patient, tumor and treatment characteristics according to existence of ACI in all 1,246 patients are listed in Table 1. Distance from anal verge to distal tumor end was a median of 2.0 cm (range, 0–3.0 cm) in the ACI group and 7.0 cm (range, 3.5–13.0 cm) in the non-ACI group. Female gender and a high histological grade were more frequent in rectal tumors with ACI. Preoperative CRT and irinotecan or an oxaliplatin-based regimen were prescribed more frequently for rectal tumors with ACI. Abdominoperineal resection was performed in 66.7% of rectal tumors with ACI. The follow-up period of all 1,246 patients was a median of 66 months (range, 3–142 months). This was not different in 189 with ACI *vs*. 1,057 without ACI; 71.4 ± 35.4 *vs*. 68.8 ± 33.1 months, respectively (*p* = 0.333).

**Table 1:** **Comparison of characteristics according to ACI in all patients**

		**With ACI (n = 192)**	**Without ACI (n = 1,054)**	***p*** **-value** ^**‡**^
Age (yr)	Mean ± SD	57.6 ± 11.3	58.2 ± 10.8	0.462
Gender	Male	114 (59.4)	718 (68.1)	0.018
	Female	78 (40.6)	336 (31.9)	
Distance from anal verge (cm)	Mean ± SD	2.0 ± 0.9	6.9 ± 2.1	<0.001
Histological grade^*^	Low	171 (90.0)	989 (96.2)	<0.001
	High	19 (10.0)	39 (3.8)	
	(Not specified = 28)			
Clinical stage	cStage II	42 (21.9)	213 (20.2)	0.599
	cStage III	150 (78.1)	841 (79.8)	
Pathological stage	ypStage 0	26 (13.5)	104 (9.9)	0.001
	ypStage I	47 (24.5)	176 (16.7)	
	pStage II/ypStage II	54 (28.1)	299 (28.4)	
	pStage III/ypStage III	65 (33.9)	475 (45.1)	
CRT timing	Preoperative CRT	168 (87.5)	709 (67.3)	<0.001
	Postoperative CRT	24 (12.5)	345 (32.7)	
Concurrent chemotherapy	Fluoropyrimidine alone	166 (86.5)	960 (91.8)	0.018
	Irinotecan or oxaliplatin-based	26 (13.5)	86 (8.2)	
	(None = 8)			
Surgery	Sphincter-sparing surgery	64 (33.3)	1,016 (96.4)	<0.001
	Abdominoperineal resection	128 (66.7)	38 (3.6)	
CRM	Negative (>0.1 cm)	154 (80.2)	890 (85.3)	0.071
	Positive (≤0.1 cm)	38 (19.8)	153 (14.7)	
	(Not specified = 11)			
Postoperative chemotherapy	Yes	175 (91.1)	962 (91.3)	0.955
	No^†^	17 (8.9)	92 (8.7)	

*Abbreviations*: *ACI* anal canal invasion, *SD* standard deviation, *CRT* chemoradiotherapy, *CRM* circumferential resection margin.

^*^Evaluated by pretreatment diagnostic biopsy. Low indicates well or moderately differentiated; high indicates poorly differentiated, mucinous, or signet ring cell carcinoma.

^†^Including patients who did not finish the planned chemotherapy.

^‡^Chi-square test, linear-by-linear association, or *t-*test.

### Patterns of IGN recurrence

Of 1,246 patients, 10 developed IGN recurrence; 7 with ACI and 3 without ACI. Characteristics of the 10 patients are listed in Table 2. All 10 patients had disease in the regional pelvic lymph nodes initially and 2 had mucinous or signet ring cell carcinoma. Distance from the anal verge to the distal tumor end was a median of 1 cm (range, 0–2.5 cm) in patients with ACI and ranged from 5–10 cm in patients without ACI. Patients’ characteristics were compared according to IGN recurrence (Table 3). Significant difference existed in the rate of sphincter-sparing surgery, in addition to tumor distance from the anal verge; however, only the tumor distance maintained its statistical significance in the multivariate analysis (≤3 cm *vs*. > 3 cm; odds ratio 7.287, 95% confidence interval 1.102 – 48.171, *p* = 0.039).

**Table 2:** **Characteristics of patients with IGN recurrence**

**No**	**ACI**	**Gender/Age**	**cStage**	**pStage/ypStage**	**Hisological grade** ^**†**^	**Surgery**
1	Yes (1.0)^*^	F/38	cT3N1M0	ypT2N1bM0	Low	LAR
2	Yes (1.5)	F/57	cT4N1M0	ypT0N0M0	High	APR
3	Yes (0.0)	M/59	cT3N1M0	ypT4bN1bM0	Low	APR
4	Yes (2.5)	M/55	cT3N2M0	ypT3N0M0	Low	APR
5	Yes (0.5)	M/50	cT3N2M0	ypT2N0M0	Low	APR
6	Yes (0.0)	M/58	cT3N1M0	ypT4bN2aM0	Low	APR
7	Yes (2.0)	F/58	cT3N2M0	pT3N2aM0	High	APR
8	No (5.0)	M/57	cT3N2M0	pT3N2aM0	Low	LAR
9	No (9.0)	M/52	cT3N2M0	pT3N2bM0	Low	LAR
10	No (10.0)	M/54	cT3N2M0	pT3N2aM0	Low	LAR

*Abbreviations*: *IGN* inguinal node, *ACI* anal canal invasion, *LAR* low anterior resection, *APR* abdominoperineal resection.

^*^Data in parenthesis are the distance (cm) from the anal verge to the distal end of the tumor.

^†^Evaluated by pretreatment diagnostic biopsy. Low indicates well or moderately differentiated; high indicates poorly differentiated, mucinous, or signet ring cell carcinoma.

**Table 3:** **Comparison of characteristics according to IGN recurrence in all patients**

		**With IGN recurrence (n = 10)**	**Without IGN recurrence (n = 1,236)**	***p*** **-value** ^**‡**^
Age (yr)	Mean ± SD	53.8 ± 6.3	58.1 ± 10.9	0.057
Gender	Male	7 (0.8)	825 (99.2)	1.000
	Female	3 (0.7)	411 (99.3)	
Distance from anal verge (cm)	Mean ± SD	3.4 ± 3.8	6.2 ± 2.6	0.046
Histological grade^*^	Low	8 (0.7)	1152 (99.3)	0.078
	High	2 (3.4)	56 (96.6)	
	(Not specified = 28)			
Clinical stage	cStage II	0	255 (100)	0.229
	cStage III	10 (1.0)	981 (99.0)	
Pathological stage	ypStage 0	1 (0.8)	129 (99.2)	0.267
	ypStage I	1 (0.4)	222 (99.6)	
	pStage II/ypStage II	1 (0.3)	352 (99.7)	
	pStage III/ypStage III	7 (1.3)	533 (98.7)	
CRT timing	Preoperative CRT	6 (0.7)	871 (99.3)	0.494
	Postoperative CRT	4 (1.1)	365 (98.9)	
Concurrent chemotherapy	Fluoropyrimidine alone	10 (0.9)	1116 (99.1)	0.613
	Irinotecan or oxaliplatin-based	0	112 (100)	
	(Not specified = 8)			
Surgery	Sphincter-sparing surgery	4 (0.4)	1076 (99.6)	0.001
	Abdominoperineal resection	6 (3.6)	160 (96.4)	
CRM	Negative (>0.1 cm)	9 (0.9)	1035 (99.1)	1.000
	Positive (≤0.1 cm)	1 (0.5)	190 (99.5)	
	(Not specified = 11)			
Postoperative chemotherapy	Yes	10 (0.9)	1127 (99.1)	1.000
	No^†^	0	109 (100)	

*Abbreviations*: *IGN* inguinal node, *SD* standard deviation, *CRT* chemoradiotherapy, *CRM* circumferential resection margin.

^*^Evaluated by pretreatment diagnostic biopsy. Low indicates well or moderately differentiated; high indicates poorly differentiated, mucinous, or signet ring cell carcinoma.

^†^Including patients who did not finish the planned chemotherapy.

^‡^Fisher’s exact test, linear-by-linear association, or *t-*test.

Actuarial IGN recurrence at 5 years was 0.7%. When estimated separately in patients with and without ACI, the rates were 3.5% and 0.2%, respectively (*p* < 0.001). The disease-free interval until IGN recurrence was a median of 29 months (range, 8–105 months) from initial management; 27.9 ± 16.5 *vs*. 50.3 ± 49.1 months with and without ACI, respectively (*p* = 0.513).

Isolated IGN recurrence occurred in three patients, all of whom had tumors with ACI. The other seven patients showed additional synchronous recurrences; both pelvic recurrence and distant metastasis in four, distant metastasis without pelvic recurrence in two, and one showed pelvic recurrence first at 93 months; this patient subsequently (1 year later) developed IGN recurrence and peritoneal seeding.

### Salvage outcomes

Salvage outcomes of the 10 patients with IGN recurrence are described in Table 4. Among the seven patients with ACI, isolated IGN recurrence occurred in three and IGN recurrence combined with pelvic recurrence and/or distant metastasis in four. All three patients with isolated IGN recurrence received curative intent local treatments. Concurrent CRT was performed in two patients (patients #1 and 2) and surgical excision followed by concurrent CRT in one (patient #3). RT target included only the involved groin region and prescribed total RT doses were 63, 72, and 54 Gy, respectively, in the three patients. Status at the last follow-up showed no evidence of disease for 10 years after salvage treatment in one patient (patient #1). Salvage treatments in the other two patients controlled successfully the IGN recurrence for >5 years, but second malignancy or disease recurrence occurred; one (patient #2) developed endometrial cancer 31 months after salvage resulting in death, and the other (patient #3) is alive but developed lung metastasis, pelvic and retroperitoneal lymph node recurrences 38 months after salvage. Four patients (patients #4–7) with ACI and non-isolated IGN recurrence received palliative chemotherapy but died of the disease at 5–22 months after IGN recurrence.

**Table 4:** **Salvage outcomes of patients with IGN recurrence**

**No**	**Isolated IGN recurrence**	**Synchronously recurrent sites**	**Laterality of IGN recurrence**	**Time to IGN recurrence (months)**	**Survival after IGN recurrence (months)**	**Last status**
1	Yes		Bilateral	8	120	NED
2	Yes		Unilateral	61	63	Dead^*^
3	Yes		Unilateral	20	61	AWD
4	No	Brain	Bilateral	29	5	DWD
5	No	LPN, PAN	Bilateral	30	21	DWD
6	No	LPN, PAN	Unilateral	28	22	DWD
7	No	PAN	Unilateral	19	11	DWD
8	No	Tumor bed, lung	Unilateral	36	20	DWD
9	No	LPN, PAN	Bilateral	10	11	DWD
10	No	LPN, seeding	Unilateral	105^†^	8	DWD

*Abbreviations*: *IGN* inguinal node, *LPN* lateral pelvic node, *PAN* para-aortic node, *NED* no evidence of disease, *AWD* alive with disease, *DWD* dead with disease.

^*^IGN recurrence was successfully salvaged with concurrent CRT, but endometrial cancer developed 31 months after IGN recurrence that progressed to lung metastasis and peritoneal seeding causing death.

^†^Recurrence developed initially in the tumor bed at 93 months and non-isolated IGN recurrence occurred 1 year later.

Non-isolated IGN recurrence occurred in all three patients without ACI. They received palliative CRT, chemotherapy, pelvic exenteration or supportive care, but died of rectal cancer at 8, 11, and 20 months after IGN recurrence.

## Discussion

In the 2009 consensus report of the Radiation Therapy Oncology Group on the definition of clinical target volume for anorectal cancer, the panel group was divided as to whether elective irradiation of the IGN region in rectal adenocarcinomas with ACI is necessary [5]. So far, few studies have focused on the optimal RT fields for rectal cancer with ACI. Taylor *et al*. [6] assessed IGN recurrence rates in rectal cancer with ACI after preoperative or postoperative pelvic RT, of which the treatment field did not include the groin region. The 5-year IGN recurrence rate was 4% (6 of 184 patients) in rectal cancer with ACI. In other patients with rectal tumors and without ACI, the 5-year IGN recurrence rate was < 1% (3 of 350 patients). Based on their observation of low recurrence rates in IGN and the historical reports of groin RT-related morbidities, the authors insisted against routine elective groin irradiation for rectal cancer with ACI. The present study analyzed a larger number of patients and reported similar IGN recurrence rates. The 5-year IGN recurrence rate was 3.5% in 189 patients with rectal tumors with ACI and 0.2% in 1,057 patients without ACI. These outcomes support the insistence that routine irradiation of clinically negative IGN in rectal cancers with ACI is difficult to be justified.

In contrast, Lee *et al*. [7] reported that elective irradiation of IGN is highly effective in controlling its subclinical disease with minimal morbidity. They performed elective IGN irradiation in patients with pelvic tumors suspected to be at significant (>10%) risk of harboring occult subclinical disease in IGN. Primary tumor sites were heterogeneous, and included the cervix, vagina, rectum (with ACI), anus, vulva, urethra, and penis. Of 164 patients treated, 105 (rectum = 22) were eligible for analysis of IGN control. IGN recurrence was not detected in rectal cancer patients but developed in three patients with vulvar cancer and one with cervical cancer; the IGN recurrence rate was 4% at 2 years. However, the authors excluded seven patients with IGN recurrence (primary not specified) from IGN control analysis because the pelvic recurrence preceded their IGN recurrences. They thought that in this situation, IGN recurrence occurred by reseeding from the pelvic recurrence, which could cause bias in the analysis. In the present study, pelvic recurrence was present with or before IGN recurrence in half (5/10) of the patients with IGN recurrence. In the lower rectum, the difference in local anatomy (limited visualization and access) and surgical technique applied (abdominoperineal resection) lead to more frequent local pelvic recurrences compared with in the mid or upper rectum [8,9]. When pelvic recurrence develops, interruption of ascending lymphatic drain due to a previous lymphadenectomy causes retrograde lymphatic flow into the IGN [10]. If these cases were excluded from our analysis using the same approach, estimated IGN recurrence risk in patients with ACI would be further decreased.

Pelvic recurrence and/or distant metastasis was detected synchronously with IGN recurrence in rectal tumors without ACI, whereas isolated IGN recurrence developed only in rectal tumors with ACI, both in the present study and that by Taylor *et al*. [6]. Salvage therapy for isolated IGN recurrence appeared to yield durable control in the IGN region. Taylor *et al*. [6] treated three rectal cancer patients with isolated IGN recurrence using groin RT and subsequent chemotherapy. All achieved local control in the groin region, although one patient subsequently developed pelvic and distant metastases. Mesko *et al*. [11] reported that the median survival of rectal cancer patients (n = 4) with isolated IGN recurrence was significantly better than that of those (n = 9) with non-isolated IGN recurrence (20 *vs*. 12 months from IGN recurrence; *p* = 0.045). In a case series, Bardia *et al*. [12] suggested that solitary IGN metastasis, either at presentation or metachronous, may represent a distinct subset of distal rectal cancer with a more favorable prognosis compared with other metastatic rectal cancers. Graham *et al*. [13] reported that isolated IGN recurrence developed only in patients whose primary rectal tumor was located within 3 cm of the anal verge and who had an earlier tumor stage at presentation compared with those who developed non-isolated IGN recurrence. All eight patients with isolated IGN recurrence underwent groin dissection; six survived more than 2 years after. In the present study, salvage therapy using concurrent CRT and/or surgical excision yielded long-term control of recurrent disease in IGN (10 years in one and >5 years in two patients; Table 3). The isolated IGN recurrence may be considered an oligometastatic state, which is increasingly regarded as an adequate indication for curative-intent local RT [14]. Our group reported favorable outcomes following definitive RT for solitary para-aortic nodal metastasis from colorectal cancer [15]. If isolated IGN recurrence represents the true recurrence risk due to the existence of ACI, and salvage of this type of recurrence is feasible, the wait-and-see policy in IGN basins for rectal cancer with ACI could be supported.

Enlargement of the RT field to encompass the groin region is associated with a variety of acute and late morbidities, such as desquamation and fibrosis of the inguinal skin, wound and bowel complications, and edema of the extremities and femoral neck fractures [6]. To avoid these morbidities, omission of prophylactic inguinal irradiation is suggested for early-stage (cT1) anal cancer, which shows a < 5% IGN failure rate when IGN irradiation is eliminated [16-19]. However, innovative RT technologies, including intensity-modulated RT and proton therapy, will reduce the exposure of normal organs to radiation and accordingly the radiation-related morbidities in the treatment of anorectal cancer [20]. Furthermore, identification of the disease characteristics linked to an increased risk of developing IGN recurrence in addition to ACI will assist in decisions regarding elective IGN irradiation in the minority of patients who will benefit from this treatment despite the potential for complications. Research in rectal cancer has included the sentinel node procedure [21], serial acquisition of ^18^F-deoxyfluoroglucose positron emission tomography-CT [22], and the study of histological tumor features at the distal front invading the dentate line [23].

## Conclusion

Although in the present study IGN recurrence from rectal cancer developed more frequently in the presence of ACI, the 5-year rate was 3.5% despite the fact that the IGN region was not irradiated. Isolated IGN recurrence occurred only from rectal tumors with ACI and increased the IGN recurrence rate of these rectal tumors; however, salvage of isolated IGN recurrence appeared feasible. Our results indicated that routine elective IGN irradiation is unnecessary for distal rectal cancer with ACI.
